# Case Report: Zero-contrast multivessel PCI with left main bifurcation guided by dual IVUS strategies using a coronary CTA roadmap

**DOI:** 10.3389/fcvm.2026.1885875

**Published:** 2026-07-22

**Authors:** Tianyu Hu, Meiping Huang, Lei Huang

**Affiliations:** 1 Department of Catheterization Lab, Guangdong Provincial People’s Hospital, Guangdong Academy of Medical Sciences, Southern Medical University, Guangzhou, China; 2 Department of Ultrasound, Guangdong Provincial People’s Hospital, Guangdong Academy of Medical Sciences, Southern Medical University, Guangzhou, China

**Keywords:** computed tomography angiography, coronary bifurcation, intravascular ultrasound, left main disease, zero-contrast PCI

## Abstract

**Background:**

Zero-contrast percutaneous coronary intervention (PCI) has emerged as an alternative strategy for patients with contraindications to iodinated contrast media. Nevertheless, the majority of reported cases depend on prior invasive coronary angiography for procedural planning, and experience using coronary computed tomography angiography (CTA) as the sole anatomical roadmap remains limited.

**Case summary:**

We present the case of a 38-year-old man admitted with non-ST-elevation myocardial infarction (NSTEMI) and complex multivessel coronary disease, in whom prior coronary computed tomography angiography (CTA) had triggered life-threatening anaphylactic shock. No subsequent invasive coronary angiography was performed; the pre-procedural roadmap was based solely on the CTA. Using intravascular ultrasound (IVUS) guidance, we successfully performed complete multivessel, zero-contrast PCI, including treatment of a left main (LM) bifurcation lesion. The right coronary artery lesion was treated with indirect IVUS guidance, marking landing zones on fluoroscopy before stent deployment. The long, ostial left anterior descending artery (LAD) lesion extending into the LM required direct IVUS guidance, with stent advancement and deployment under continuous live IVUS imaging. Post-procedural IVUS confirmed optimal expansion and apposition with no complications. The patient remained free of cardiovascular events through 24 months of follow-up.

**Conclusion:**

Zero-contrast PCI guided by IVUS using a pre-procedural coronary CTA roadmap may be feasible in selected patients without prior invasive coronary angiography. Combining indirect and direct IVUS guidance may aid in managing complex lesions, which warrants further validation.

## 1 Introduction

Zero-contrast PCI guided by intravascular ultrasound (IVUS) is an important option for patients with contrast contraindications (1–3). Although feasible, it traditionally depends on invasive angiography for planning (4). Reports using coronary CT angiography (CTA) as the sole roadmap are scarce (1, 5). Notably, there is a lack of reports on the combined application of distinct IVUS strategies, each tailored to different lesion complexities, within a single multivessel procedure. We describe a patient in whom invasive angiography was precluded by life-threatening anaphylaxis following coronary computed tomography angiography (CTA). Using the CTA images as the sole pre-procedural roadmap, complete multivessel PCI—including left main (LM) bifurcation—was achieved under two distinct IVUS guidance strategies, entirely without contrast.

## 2 Case report

**Figure 1:**
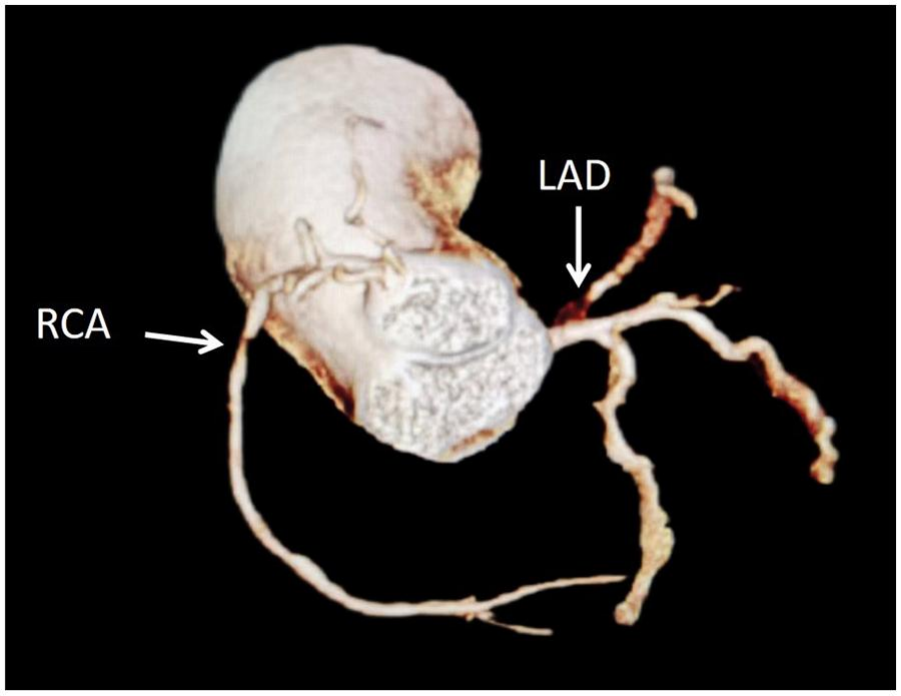
Pre-procedural coronary computed tomography angiography (CTA) serving as the sole anatomical roadmap. The CTA, obtained from an external hospital, shows severe stenosis in the proximal right coronary artery (RCA, arrow) and significant disease involving the left main (LM) and left anterior descending (LAD) artery bifurcation (arrowhead). This image served as the only pre-procedural roadmap for the zero-contrast PCI.

A 38-year-old man was admitted with non-ST-elevation myocardial infarction (NSTEMI) after a one-week exacerbation of a six-month history of exertional chest pain. One week before admission, coronary computed tomography angiography (CTA) at an outside facility, using an unspecified iodinated contrast agent, precipitated immediate anaphylactic shock with loss of consciousness and cardiac arrest, requiring emergency cardiopulmonary resuscitation. The specific iodinated contrast agent could not be identified, and no subsequent allergy testing was performed given the severity of the reaction. The CTA images were obtained 7 days prior to the index PCI procedure. Only reconstructed images were available from the referring hospital, as the original DICOM dataset could not be obtained. The image quality was sufficient to delineate vessel anatomy and lesion location, and the CTA report indicated no severe coronary calcification. No dedicated three-dimensional reconstruction software was used for procedural planning. According to the CTA report, there was severe proximal right coronary artery (RCA) stenosis and significant disease involving the left main-left anterior descending (LM-LAD) bifurcation. The reconstructed CTA images were later used intra-procedurally as the anatomical roadmap (Figure 1).

Past medical history included type 2 diabetes mellitus, and long-term tobacco use, with no documented history of hypertension. On admission, vital signs were stable (blood pressure 113/69 mmHg, heart rate 69 bpm, respiratory rate 20/min, temperature 36.5 ℃). Physical examination was unremarkable. Baseline laboratory results were within normal ranges, except for a mild elevation of high-sensitivity cardiac troponin T (15 ng/L, reference < 14 ng/L). B-type natriuretic peptide (BNP) was 67.3 pg/mL (within normal range). Echocardiography revealed a left ventricular ejection fraction (LVEF) of 55%, with hypokinesis of the mid-anterior wall and apical septum.

Due to persistent angina on optimal medical therapy and confirmed multivessel disease, coronary revascularization was indicated. The patient declined coronary artery bypass grafting (CABG). Given the history of contrast-induced cardiac arrest, a strict zero-contrast strategy was adopted. A multidisciplinary heart team therefore recommended zero-contrast PCI guided exclusively by IVUS, using the prior CTA as an anatomical roadmap. Dual antiplatelet therapy with aspirin (100 mg daily) and clopidogrel (300 mg loading dose) was initiated. No prophylactic anti-allergy medication was administered, as the procedure was planned as an absolute zero-contrast intervention. Emergency resuscitation drugs were prepared, and a small volume of low-osmolar contrast (iohexol, 50 mL) was kept as a last-resort bailout option for immediately life-threatening complications (e.g., no-reflow/slow-flow), together with intravenous dexamethasone (10 mg).

Indirect IVUS guidance was defined as pre-deployment IVUS imaging to mark distal and proximal landing zones on fluoroscopy, followed by stent delivery and deployment under fluoroscopic guidance. Direct IVUS guidance, also described as the “live IVUS stenting technique” (6), was defined as stent advancement and deployment under continuous intravascular ultrasound visualization, without relying on pre-marked fluoroscopic landing zones.

### 2.1 IVUS-guided zero-contrast PCI for RCA

Vascular access was obtained via the right femoral artery, and systemic anticoagulation was achieved with intravenous heparin (7,000 IU). A 7F SAL 1.0 guiding catheter (Medtronic) was engaged at the RCA ostium under fluoroscopic guidance. Engagement was confirmed by tactile feedback as the catheter entered the ostium, synchronous tip motion with the cardiac cycle, and continuous arterial pressure waveform monitoring without damping. A SION guidewire was advanced to the distal RCA without contrast administration.

**Figure 2:**
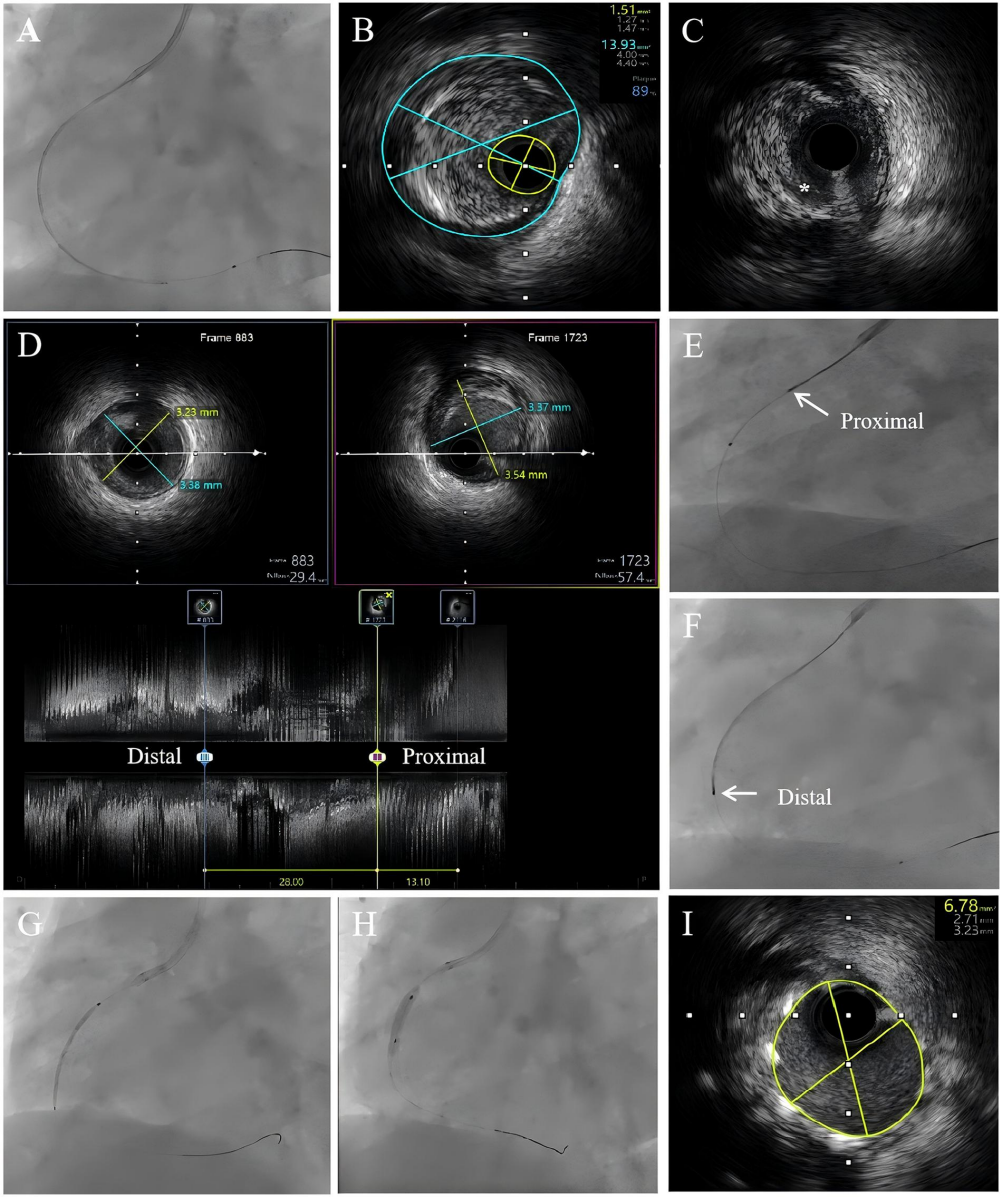
Zero-contrast RCA PCI using indirect IVUS guidance. **(A)** IVUS catheter positioned in the distal RCA before pullback. **(B)** Pre-intervention IVUS image at the most severe stenosis revealed an eccentric, echo-attenuated fibro-lipidic plaque, with a minimum lumen area (MLA) of 1.81 mm^2^ and a plaque burden (PB) of 85%. **(C)** Post-dilatation IVUS showing a significant dissection (*) at the lesion site, confirming the need for stenting. **(D)** IVUS measurements guided device selection: reference diameters (distal: 3.23 × 3.38 mm; proximal: 3.37 × 3.54 mm) and lesion length (≥28 mm). A 13-mm disease-free proximal segment (brackets) was identified as the proximal landing zone. **(E,F)** Establishing fluoroscopic landmarks. The IVUS transducer was positioned at the distal **(E)** and proximal **(F)** landing zones. The corresponding fluoroscopic images were saved, marking these positions (arrows) for subsequent stent deployment. **(G)** Fluoroscopy showing the 3.0 × 30 mm drug-eluting stent positioned using the saved landmarks. **(H)** Postdilation with a 3.0 × 15 mm noncompliant balloon. **(I)** Final IVUS confirmed optimal stent expansion [minimum stent area (MSA) 6.89 mm^2^] and apposition.

Initial IVUS pullback from the distal RCA demonstrated diffuse atherosclerotic disease, with the most severe stenosis located in the proximal segment, characterized by an eccentric fibro-lipidic plaque [minimum lumen area [MLA] 1.81 mm^2^, plaque burden [PB] 85%] (Figures 2A,B). Predilation was performed using a 2.5 × 16 mm balloon at 12–16 atm. Repeat IVUS revealed improved luminal dimensions but identified a focal dissection at the treated segment, confirming the need for stent implantation (Figure 2C).

IVUS measurements guided stent selection, with distal and proximal reference diameters of 3.23 × 3.38 mm and 3.37 × 3.54 mm, respectively, and an estimated lesion length ≥28 mm. A 13-mm relatively disease-free proximal segment was identified as a safe landing zone to minimize geographic miss (Figure 2D). Using indirect IVUS guidance, distal and proximal landing zones were marked on fluoroscopy (Figures 2E,F), and a 3.0 × 30 mm drug-eluting stent was deployed at 16 atm, followed by postdilation with a 3.0 × 15 mm noncompliant balloon at 24–28 atm (Figures 2G,H).

Final IVUS confirmed optimal stent expansion with a minimum stent area (MSA) 6.89 mm^2^, complete strut apposition, and absence of edge dissection, confirming procedural success without contrast angiography (Figure 2I).

### 2.2 IVUS-guided zero-contrast PCI for LAD and LM bifurcation

**Figure 3:**
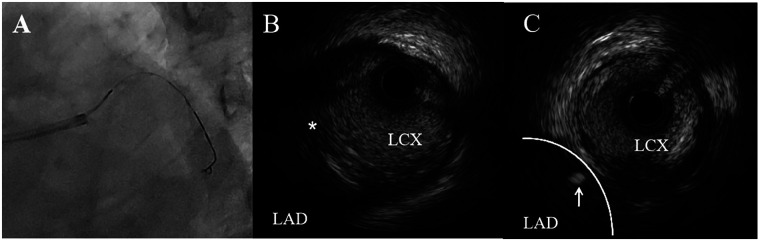
Direct IVUS guidance for wiring the ostial LAD. **(A)** IVUS catheter placed in the LCx via automated pullback from the LCx to the LM, using the LCx as an imaging window to visualize the LAD ostium. **(B)** IVUS image confirming a critical ostial LAD stenosis with a slit-like residual lumen (*). **(C)** Under real-time IVUS visualization (providing a “live” view of the ostium), a guidewire (arrow) was successfully navigated through the residual lumen into the distal LAD with microcatheter support.

A 7F SPB 3.75 guiding catheter (Asahi Intecc) was engaged in the LM coronary artery using the same stepwise approach, with CTA-derived ostial anatomy guiding fine catheter positioning, and confirmed by tactile feedback and pressure waveform monitoring. No contrast test injection was performed. Given the severe ostial LAD stenosis on CTA, a SION guidewire was first advanced into the distal LCx. IVUS pullback from the LCx to the LM enabled visualization of the LAD ostium, which was confirmed to be critically narrowed with only a slit-like residual lumen (Figures 3A,B).

**Figure 4:**
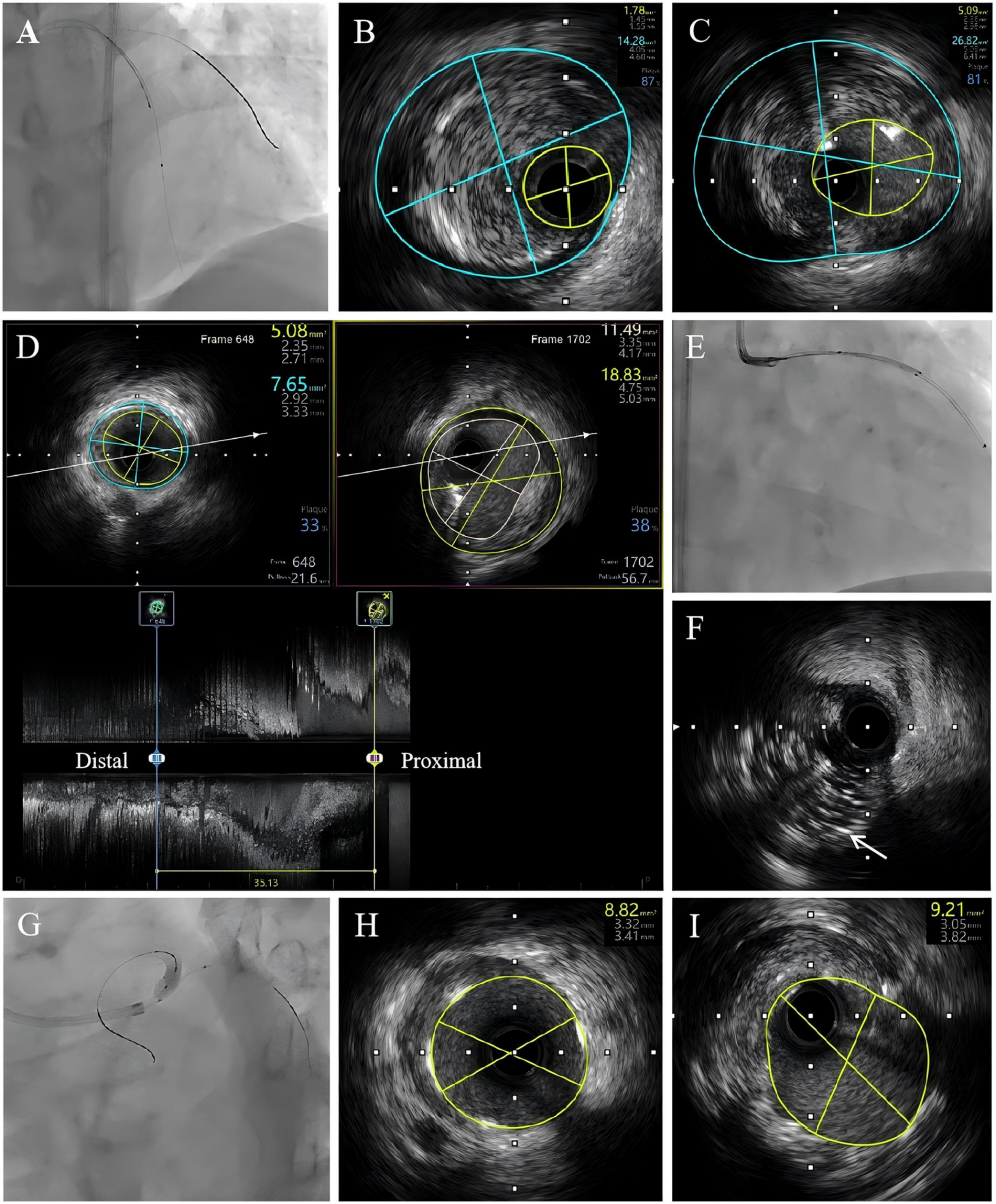
Complex LM-LAD bifurcation stenting using direct IVUS guidance. **(A)** IVUS catheter positioned in the distal LAD for pullback. **(B,C)** Pre-stenting IVUS confirmed **(B)** critical ostial LAD stenosis (MLA 1.78 mm^2^, PB 87%) and **(C)** significant plaque burden in the LM (MLA 5.09 mm^2^, PB 81%). **(D)** IVUS quantification revealed a caliber mismatch between the proximal LAD (2.92 × 3.33 mm) and LM (4.75 × 5.03 mm), necessitating two sequential stents (total length: 35 mm). **(E,F)** Direct, live IVUS-guided stenting. **(E)** The IVUS transducer was positioned at the distal landing zone. The first stent (3.0 × 18 mm) was advanced under live IVUS monitoring—without relying on pre-marked fluoroscopic landmarks—until its distal marker aligned with the transducer. **(F)** Real-time IVUS image confirmed the stent's position at the landing zone immediately prior to deployment. **(G)** After implanting the second sequential stent (3.5 × 19 mm), the bifurcation was optimized with final kissing balloon inflation (LCx: 2.0 × 20 mm; LM-LAD: 3.5 × 12 mm). **(H,I)** Final IVUS confirmed excellent expansion and apposition in the LAD (MSA 8.82 mm^2^) **(H)** and LM (MSA 9.21 mm^2^) **(I)**, with a widely patent LCx ostium.

Under real-time IVUS guidance with Finecross microcatheter support, a SION blue guidewire was successfully advanced into the distal LAD (Figure 3C), followed by predilation of the ostial LAD using a 2.5 × 16 mm balloon at 12–16 atm. Subsequent IVUS pullback from the distal LAD demonstrated diffuse LAD disease (MLA 1.78 mm^2^, PB 87%) extending into the LM (MLA 5.09 mm^2^, PB 81%), while the LCx ostium was free of significant disease (Figures 4A–C). The lesion was classified as Medina (1,1,0), favoring a provisional crossover strategy.

Marked vessel diameter mismatch between the proximal LAD (2.92 × 3.33 mm) and the LM (4.75 × 5.03 mm) necessitated implantation of two sequential overlapping stents, with an estimated total stent length of 35 mm (Figure 4D). The distal landing zone was selected within a relatively healthy LAD segment to minimize geographic miss.

Direct IVUS guidance was used for stent implantation. A 3.0 × 18 mm drug-eluting stent was deployed in the distal LAD under continuous IVUS visualization (Figure 4E,F), followed by a second 3.5 × 19 mm stent implanted from the proximal LAD into the LM with minimal (1–2 mm) overlap. The LCx was rewired, and final kissing balloon inflation was performed (LM–LAD: 3.5 × 12 mm; LCx: 2.0 × 20 mm) (Figure 4G).

Final IVUS confirmed optimal expansion (MSA 8.82 mm^2^ in the LAD and 9.21 mm^2^ in the LM), complete apposition, and no compromise of the LCx ostium (Figures 4H,I).

The total procedural time was 100 min, fluoroscopy time 38 min, cumulative radiation dose 1,912 mGy air kerma, and dose-area product (DAP) 98.7 Gy·cm^2^. Post-procedural echocardiography revealed no pericardial effusion.

At 24 months, the patient was asymptomatic, without recurrent angina, rehospitalization, myocardial infarction, or target vessel revascularization.

A detailed timeline of the clinical course is provided as Supplementary Table S1.

## 3 Discussion

To the best of our knowledge, this is the first reported case in which coronary CTA alone served as the anatomical roadmap for complex multivessel PCI, including LM bifurcation, performed entirely under IVUS guidance with systematic integration of both indirect and direct IVUS strategies. This case highlights the feasibility of IVUS as the sole intra-procedural imaging modality when invasive coronary angiography and contrast administration are contraindicated, and illustrates how combining indirect and direct IVUS guidance can facilitate precise stent deployment in challenging anatomy. A summary of previously reported zero-contrast or ultra-low contrast IVUS-guided PCI cases is provided in Supplementary Table S2, which supports the novelty of this dual-guidance approach (1, 4, 6–8).

CABG was recommended as the guideline-directed strategy for this young patient with left main disease but was declined. Medical therapy alone was insufficient for ongoing NSTEMI with multivessel disease, leaving PCI as the only revascularization option. Conventional PCI was contraindicated by the prior contrast-induced cardiac arrest. After excluding alternative strategies—gadolinium-based angiography (21% severe complication risk) (9) and premedication protocols (breakthrough anaphylaxis 1.2%–46%) (10, 11)—zero-contrast IVUS-guided PCI using the pre-procedural CTA roadmap was unanimously recommended and accepted by the patient.

Previous reports and consensus documents (6, 12–15) support the role of IVUS in ultra-low/zero-contrast PCI, including lesion assessment, procedural planning, and post-procedural optimization. Pre-procedurally, IVUS enables accurate lesion assessment by characterizing stenosis severity and morphology, confirming critical LAD involvement and ruling out LCx ostial stenosis. In procedural planning, precise IVUS measurements, including vessel dimensions and lesion length, guided the decision to use two sequential stents to address a marked caliber mismatch between the distal LAD and LM. In bifurcation strategy, IVUS defines plaque distribution and Medina classification, which in this case supported a provisional crossover approach for a Medina (1,1,0) lesion. Post-procedurally, IVUS ensures optimal stent expansion and apposition, confirming procedural success without angiographic confirmation. Procedural success was defined according to the 2018 EAPCI expert consensus criteria for IVUS-guided stent optimization: MSA ≥ 8.0 mm^2^ for the proximal LMCA and ≥ 6.0 mm^2^ for the LAD ostium (the ‘5-6-7-8 rule’), with complete strut apposition and no edge dissection (15). The achieved MSA values (9.21 mm^2^ in the LM, 8.82 mm^2^ in the LAD) substantially exceeded these thresholds.

A comparison of the practical indications, advantages, and limitations of indirect vs. direct IVUS guidance is summarized in Supplementary Table S3. The procedural strategy effectively leveraged the complementary roles of indirect and direct IVUS guidance. Indirect IVUS guidance relies on pre-procedural IVUS imaging to mark optimal stent landing zones on fluoroscopy before device delivery (16). This method is best suited for relatively straight vessel segments with generous landing zones proximal and distal to the lesion (7). It offers procedural simplicity, reduces catheter manipulation, and shortens procedure time (17). However, accuracy may be affected by cardiac or respiratory motion, and in curved vessels, stent delivery may slightly stretch or deform the vessel, increasing the risk of geographic miss. In such situations, selecting a longer stent can help mitigate this risk (7, 16). Direct IVUS guidance permits continuous visualization of stent advancement and deployment within the vessel, enabling precise placement in diffusely diseased or anatomically complex segments. It is particularly valuable when accurate positioning is required in tortuous or long lesions. Limitations include the need for a larger guiding catheter to accommodate both the IVUS probe and stent system, and a slightly higher risk of technical issues such as catheter entrapment during withdrawal (6). In this case, indirect IVUS was used for the RCA, where the vessel was relatively straight and a generous stent landing zone was available, ensuring efficiency without compromising accuracy. For the LAD-LM bifurcation, direct IVUS was employed, allowing precise stent placement in the long, diffuse disease zone. Tailoring the choice of IVUS technique to the lesion characteristics optimized procedural outcomes, leveraging the simplicity of indirect guidance and the precision of direct guidance, effectively minimizing the limitations of each method.

The procedural radiation dose (air kerma 1,912 mGy; DAP 98.7 Gy·cm^2^) in this complex multivessel zero-contrast PCI falls within the range reported for other IVUS-guided complex interventions. Karacsonyi et al. reported a median air kerma of 1.78 Gy (IQR 1.00–3.09) for IVUS-guided CTO-PCI (18), and other series of zero-contrast IVUS-guided PCI have reported mean radiation doses ranging from 0.94 Gy to 2.93 Gy (19, 20). Our value of 1.91 Gy is therefore consistent with the expected range for procedures of this complexity. With increasing operator experience, further dose optimization is anticipated.

Routine invasive angiography and coronary CTA were avoided due to contrast hypersensitivity. CT with desensitization was not pursued: the triggering contrast agent was unknown, and breakthrough anaphylaxis remains possible despite premedication (reported rates 1.2%–46%) (10, 11) —an unacceptable risk after prior contrast-induced cardiac arrest. Gadolinium-based angiography carries a 21% risk of severe complications (9) and was therefore excluded. Non-invasive stress imaging was deferred as the patient remained asymptomatic with preserved left ventricular function during 24-month follow-up. The absence of systematic follow-up imaging is acknowledged as a limitation.

In this case, IVUS-guided zero-contrast PCI was completed successfully using a pre-procedural coronary CTA roadmap, despite the absence of prior invasive coronary angiography. The tailored combination of indirect and direct IVUS guidance allowed the operator to manage different lesion types within a single complex multivessel PCI session.

This approach may be feasible in selected high-risk patients at experienced centers, but as a single case, the findings do not permit conclusions regarding generalizability—particularly to less experienced operators or patients with more complex anatomy. Pre-procedural and post-procedural angiography were precluded by contrast contraindication. All procedural decisions therefore relied on IVUS, which provides quantitative lesion assessment, confirms stent expansion and apposition, and detects complications (dissection, hematoma, thrombus)—though assessment of small side branches and the distal vessel flow remains limited. Furthermore, follow-up relied on clinical outcomes alone, as anatomical or functional imaging was not performed. Long-term vessel patency and the absence of restenosis therefore cannot be objectively confirmed. These are acknowledged as inherent limitations. Further studies are needed to assess safety, reproducibility, and applicability in broader clinical practice.

## 4 Conclusion

IVUS-guided zero-contrast PCI using a pre-procedural coronary CTA roadmap, with combined indirect and direct IVUS guidance, may be feasible in carefully selected patients with complex multivessel disease, including LM bifurcation lesions, when prior invasive coronary angiography is unavailable. This dual-guidance strategy may help address varied lesion characteristics while maintaining procedural safety. Further research is required to verify reproducibility before wider clinical adoption.

## Data Availability

The original contributions presented in the study are included in the article/Supplementary Material, further inquiries can be directed to the corresponding author.
