# Supplementary material for: Case Report: Zero-contrast multivessel PCI with left main bifurcation guided by dual IVUS strategies using a coronary CTA roadmap
Source: Front Cardiovasc Med. 2026 Jul 22;13:1885875. doi: 10.3389/fcvm.2026.1885875 (PMC13437360; doi:10.3389/fcvm.2026.1885875)
Supplement: Supplementary file 1 [file Table1.docx]

Supplementary Material

# 1 Supplementary Table

**Supplementary Table S1. Timeline of the clinical course from symptom onset through 24-month follow-up.**

| Time point | Key event |
| --- | --- |
| 6 months prior to admission | Onset of exertional chest pain |
| 1 week prior to admission | Coronary CTA performed at an outside facility; anaphylactic shock immediately after iodinated contrast administration requiring emergency resuscitation |
| Day of admission | Diagnosed with NSTEMI; hs-cTnT 15 ng/L (reference <14 ng/L); LVEF 55% with hypokinesis of mid-anterior wall and apical septum |
| During hospitalization | Multidisciplinary heart team recommended zero-contrast PCI guided by IVUS using prior CTA as anatomical roadmap; CABG declined by patient |
| Day of PCI (total procedural time: 100 min) | RCA lesion treated with indirect IVUS guidance (3.0×30 mm DES); LM-LAD bifurcation lesion treated with direct IVUS guidance (3.0×18 mm + 3.5×19 mm sequential DES); final kissing balloon inflation; final IVUS confirmed optimal expansion and apposition |
| Immediately post-PCI | Echocardiography revealed no pericardial effusion |
| 24-month follow-up | Asymptomatic; no recurrent angina, rehospitalization, myocardial infarction, or target vessel revascularization |

Abbreviations: CABG, coronary artery bypass grafting; CTA, computed tomography angiography; DES, drug-eluting stent; hs-cTnT, high-sensitivity cardiac troponin T; IVUS, intravascular ultrasound; LAD, left anterior descending artery; LM, left main; LVEF, left ventricular ejection fraction; NSTEMI, non-ST-elevation myocardial infarction; PCI, percutaneous coronary intervention; RCA, right coronary artery.
