# Supplementary material for: Case Report: Zero-contrast multivessel PCI with left main bifurcation guided by dual IVUS strategies using a coronary CTA roadmap
Source: Front Cardiovasc Med. 2026 Jul 22;13:1885875. doi: 10.3389/fcvm.2026.1885875 (PMC13437360; doi:10.3389/fcvm.2026.1885875)
Supplement: Supplementary file 2 [file Table2.docx]

Supplementary Material

# 1 Supplementary Table S2

## **Supplementary Table S2. Previously reported zero-contrast / ultra-low contrast IVUS-guided PCI cases**

| Author, Year | Lesion Characteristics | Pre-procedural Roadmap | IVUS Guidance | Follow-up | Novelty & Distinction from Present Case |
| --- | --- | --- | --- | --- | --- |
| Lichaa, 2021 | Focal RCA lesion (case report, proof of concept) | Low-contrast angiography | Direct (live IVUS stenting) | Not reported | First systematic description of live IVUS stenting technique |
| Kumar et al., 2021 | 15 patients (mean age 70, 27 vessels), 4 LM | Prior invasive angiography | Indirect | 3 months | Zero-contrast LM bifurcation PCI |
| Moretti et al., 2023 | Multivessel + LM bifurcation + heavy calcification (76M) | Ultra-low contrast angiography | Indirect | Not reported | Multivessel + LM bifurcation; but ~30 mL contrast used |
| Sakellariou et al., 2024 | Mixed (including LM, CTO); 4 patients | Prior invasive angiography | Indirect | 3–12 months | First case series |
| Wang et al., 2026 | LAD–LM (50M, NSTE-ACS); RCA +LAD | MRCA (zero-contrast) | Indirect | 1 month | First MRCA-guided PCI |
| **Present case** | **Multivessel (RCA + LM-LAD bifurcation) (38M)** | **CTA (no prior angiography)** | **Dual (indirect for RCA + direct for LM-LAD)** | **24 months** | **CTA-guided; dual IVUS strategies; long follow-up** |

Abbreviations: CTA, computed tomography angiography; CTO, chronic total occlusion; LAD, left anterior descending; LM, left main; MRCA, magnetic resonance coronary angiography; NSTE-ACS, non–ST-elevation acute coronary syndrome; NSTEMI, non–ST-elevation myocardial infarction; PCI, percutaneous coronary intervention; RCA, right coronary artery.
