# Supplementary material for: Case Report: Zero-contrast multivessel PCI with left main bifurcation guided by dual IVUS strategies using a coronary CTA roadmap
Source: Front Cardiovasc Med. 2026 Jul 22;13:1885875. doi: 10.3389/fcvm.2026.1885875 (PMC13437360; doi:10.3389/fcvm.2026.1885875)
Supplement: Supplementary file 3 [file Table3.docx]

Supplementary Material

# 1 Supplementary Table S3

**Supplementary Table S3. Practical indications, advantages, and limitations of indirect versus direct IVUS guidance**

|  | Indirect IVUS Guidance | Direct IVUS Guidance |
| --- | --- | --- |
| Indications | Straight vessels with generous landing zones | Tortuous, diffusely diseased, or ostial lesions; when precise stent positioning is critical |
| Advantages | Procedural simplicity; less catheter manipulation; shorter procedure time | Real-time verification of stent position; reduces geographic miss in complex anatomy |
| Limitations | Accuracy affected by motion; risk of geographic miss | Requires larger guiding catheter; risk of catheter entrapment |
